# Computationally Efficient Impact Estimation of Coil Misalignment for Magnet-Free Cochlear Implants

**DOI:** 10.3390/s25144379

**Published:** 2025-07-13

**Authors:** Samuelle Boeckx, Pieterjan Polfliet, Lieven De Strycker, Liesbet Van der Perre

**Affiliations:** 1 KU Leuven Ghent, Campus Rabot, 9000 Ghent, Belgium; 2 Cochlear Limited, Cochlear Technology Centre Belgium, 2800 Mechelen, Belgium

**Keywords:** coupling coefficient, misalignment, cochlear implant, MATLAB implementation

## Abstract

A cochlear implant (CI) system holds two spiral coils, one external and one implanted. These coils are used to transmit both data and power. A magnet at the center of the coils ensures proper alignment to assure the highest coupling. However, when the recipient needs a magnetic resonance imaging (MRI) scan, this magnet can cause problems due to the high magnetic field of such a scan. Therefore, a new type of implant without magnets would be beneficial and even supersede the current state of the art of hearing implants. To examine the feasibility of magnet-free cochlear implants, this research studies the impact of coil misalignment on the inductive coupling between the coils and thus the power and data transfer. Rather than using time-consuming finite element analysis (FEA), MATLAB is used to examine the impact of lateral, vertical and angular misalignment on the coupling coefficient using derivations of Neumann’s equation. The MATLAB model is verified with FEA software with a median 8% relative error on the coupling coefficient for various misalignments, ensuring that it can be used to study the feasibility of various magnet-free implants and wireless power and data transmission systems in general. In the case of cochlear implants, the results show that by taking patient and technology constraints like skinflap thickness and mechanical design dimensions into account, the mean error can even be reduced to below 5% and magnet-free cochlear implants can be feasible.

## 1. Introduction

Cochlear implants (CI) are used for people with severe to profound hearing loss due to a damaged inner ear. In current systems, there is an implanted and an external part of the CI system, and both hold a spiral coil for the transmission of the signal and power. The alignment of both coils is assured by a magnet at the center of these coils. Having a magnet implanted raises some concerns like the size of the implant and magnetic resonance imaging (MRI) compatibility. Therefore, this research investigates the challenges that come with magnet-free cochlear implants. The alignment of the coils is a key challenge in order to assure a quality hearing experience. To check the consequences of this misalignment and potentially smaller designs, COMSOL Multiphysics (COMSOL Multiphysics version 6.1) is used to simulate the coils. This simulation software, using finite element analysis (FEA), can be very accurate but becomes rather time-consuming when dealing with complex models. It can take hours, or up to days. A software tool to quickly indicate if the value of the coupling coefficient between the two coils is still acceptable would significantly speed up the design process. This paper describes the development of a MATLAB-based (Mathworks, Inc. MATLAB version R2023a) framework that allows computationally efficient estimation of the impact of the misalignment between two coils on the coupling coefficient. This framework has a broader application range than cochlear implants alone. It can be used to design coils for all sorts of medical implants or loosely coupled charging solutions where misalignment is likely, such as unmanned aerial vehicle (UAV)-based charging of Internet-of-Things (IoT) nodes [1,2] or electrical vehicle (EV) charging [3,4].

This paper is organized as follows: the remainder of this section describes the functioning of a cochlear implant and the challenges this research entails. The following section studies the literature and proposes a methodology to calculate the coupling coefficient. Section 3 validates the results and, finally, a discussion and conclusion follow in Section 4 and Section 5, respectively.

### 1.1. A Cochlear Implant

**Figure 1:**
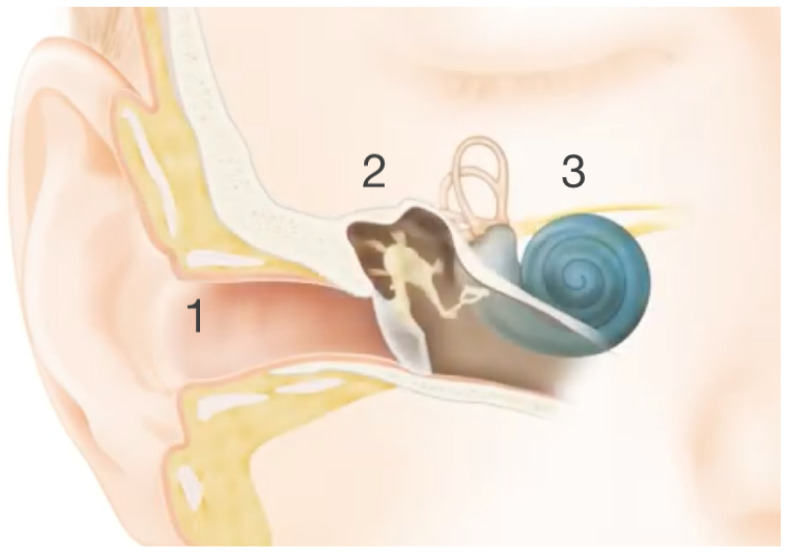
The three parts of the ear: the outer ear (1), the middle ear (2) and the inner ear (3). Cochlear™ [6].

The ear consists of three parts: the outer ear, the middle ear and the inner ear. The outer ear consists of the pinna and the ear canal; see number 1 on Figure 1. These parts pick up sound waves and send them to the eardrum, which is part of the middle ear together with three tiny bones called the hammer, anvil and stirrup; see number 2 on Figure 1. The eardrum vibrates due to the sound waves and puts the bones into motion. The inner part of the ear consists of the cochlea and the hearing nerve; see number 3 on Figure 1. Fluid in the cochlea transfers the motion of the bones towards hair cells that absorb the movement, creating electrical impulses that are sent to the brain through the hearing nerve. The brain converts these impulses into sound [5].

When one of those three parts does not function properly, hearing loss might be experienced. Different types of hearing loss are defined depending on which part of the ear is failing. Different types have different solutions. A CI is recommended in the case of sensorineural hearing loss. This type occurs when there is damage to the inner ear, namely the cochlea. The result is that sounds are more silent and harder to understand. When the hearing loss is only mild to moderate, a hearing aid might suffice; however, for severe to profound hearing loss, a cochlear implant is the most effective. The difference is that hearing aids amplify sounds while cochlear implants bypass the damaged part and directly send impulses to the hearing nerve [7].

**Figure 2:**
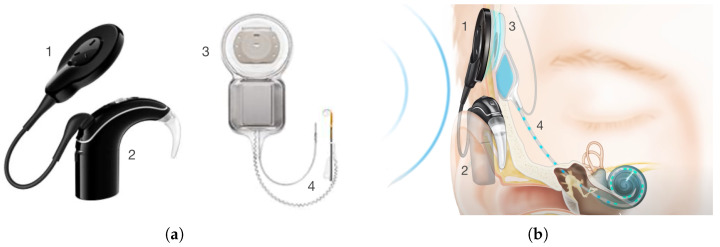
A cochlear implant with the external coil (1), the BTE (2), the implanted coil (3) and the electrodes (4). Cochlear™. (**a**) A cochlear implant system [9]. (**b**) Once implanted [10].

A cochlear implant system consists of two parts: an external and an implanted part. The external part consists of two smaller parts; a part called the BTE (behind-the-ear), consisting of a sound processor, microphone and a battery and a part that holds a coil and a magnet. The implantable part consists of a coil, magnet and electrodes. The different parts can be seen in Figure 2a. The implant position after implantation (between the skin and the skull) can be seen in Figure 2b. The functioning of the cochlear implant is as follows: sound waves are picked up by microphones in the BTE. The processor filters and converts these waves into digital information and sends this information to the external coil. Due to the magnetic coupling between the two coils, the signal and also the power from the battery are transferred to the internal coil. To assure the best transfer, the alignment of these coils is very important. Therefore, magnets are placed at the centers. The implant converts the signal it receives from the external coil to electrical impulses to send through the electrodes to the cochlea. Here, the signal is picked up by the hearing nerve and sent to the brain, resulting in sound [8].

### 1.2. Challenge

When the first cochlear implants were developed, MRI was only used on the brain. Nowadays the technology has advanced towards a widely available routine relied upon by different specialists to evaluate the heart, the knees, the spine, the abdomen, etc. It is thus likely that an MRI is recommended at some point in life. This creates a problem for CI recipients, seeing that an MRI scan induces a magnetic field with a magnetic flux density of around 1.5 T to 3 T [11]. This is very large compared to the magnetic field of the earth which is around 0.05 mT [12]. As a result of this high magnetic flux density, the magnet at the center of the implanted coil may start to move, depending on the type of implant and its manufacturer and generation. This might cause uncomfortable and even painful situations for the patient [13,14,15]. A solution is to use a headband to prevent the magnet from moving, however, this can still cause some discomfort and the magnet can distort the magnetic field of the scanner, causing the images to be of poor quality. Another solution is to perform surgery to remove the magnet and again after taking the images, to put it back. However, this solution makes the small procedure of an MRI scan a cumbersome operation [16].

**Figure 3:**
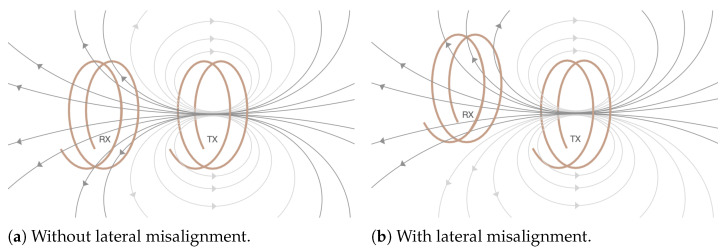
Flux linkage.

The ideal situation would be that there is no magnet at all. The implant would consist of just a coil and the electrodes. The issue here is that the alignment of the two coils cannot be guaranteed anymore. As seen in Figure 3, less flux lines are caught by the secondary coil in the case of misalignment. As a result the quality of the transmitted signal and the amount of power transferred will be reduced.

Without the magnet, there is an extra advantage, namely that the coils can be smaller and thus less visible, seeing that there is nothing taking up space at the center anymore. With smaller coils, however, the impact of misalignment is even greater. This research examines the effects of misalignment on the coupling coefficient between the two coils.

## 2. Materials and Methods

This section describes different approaches found in the literature to calculate the coupling coefficient between the two coils. The goal is to find a method that allows efficient computation in MATLAB and that gives a first estimation of the coupling coefficient to speed up the coil design process in early stages of development. The following subsections describe formulas of the coupling coefficient, and the self-inductance of a coil and the mutual inductance between two coils which are necessary to calculate this coupling coefficient. Different approaches can be found in the literature to find the mutual inductance, resulting in different computation times. Finally, one approach results in a MATLAB implementation.

### 2.1. The Coupling Coefficient

The coupling coefficient *k* is determined as Equation (1) [17]:

(1) $$k=\frac{M}{\sqrt{L_{p}L_{s}}}$$

where *M* is the mutual inductance between the two coils, $L_{p}$ the self-inductance of the primary coil and $L_{s}$ the self-inductance of the secondary coil. The coupling coefficient represents the flux density captured by the secondary coil compared to flux density induced by the primary coil. It is a fractional value of the total flux linkage possible. If *k* is zero, there is no inductive coupling. If *k* is one, the two coils are perfectly coupled, i.e., power transfer without any losses.

The coupling coefficient thus indicates to what extent signal and power are being transferred. To determine *k*, only the self-inductance of each coil and the mutual inductance between them are required.

#### 2.1.1. The Self-Inductance of a Coil

To calculate the self-inductance of a coil, the inductance of each winding needs to be calculated, followed by the mutual inductance between all windings. The sum of these values results in the self-inductance of the coil. The inductance of one circular loop is given by the following formula [18]:

(2) $$L=\mu_{0}\mu_{rm}r\left(ln(\frac{8r}{a})-1.75\right)$$

**Figure 4:**
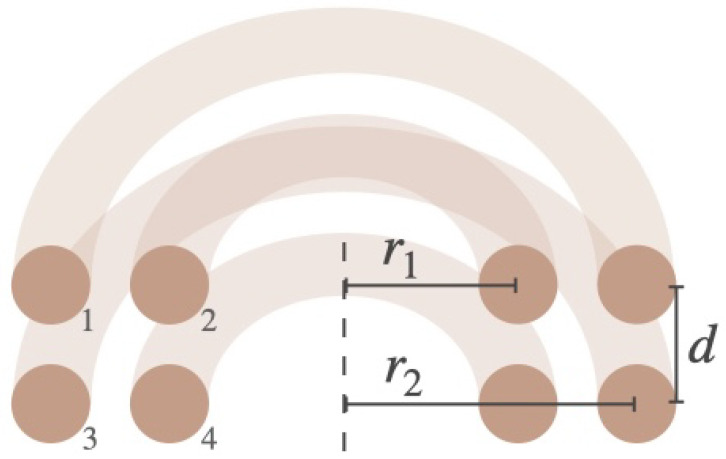
Cross section of a coil with four turns distributed over two layers.

The mutual inductance between two windings of one and the same coil is calculated using following formula

(3) $$M=\mu_{0}\;\sqrt{r_{1}r_{2}}\left[\left(\frac{2}{s}-s\right)K\left(s\right)-\frac{2}{s}E\left(s\right)\right]$$

where

(4) $$s=\sqrt{\frac{4r_{1}r_{2}}{{\left(r_{1}+r_{2}\right)}^{2}+d^{2}}}$$

where $r_{1}$ is the radius of one winding, $r_{2}$ the radius of the other winding, *d* the vertical distance between the centers of the wires, as shown in Figure 4, and K(s) and E(s) are the complete elliptic integrals of the first and second kind, respectively [19]:

(5) $$K\left(s\right)=\int_{0}^{\frac{\pi }{2}}\frac{1}{\sqrt{1-s^{2}\sin^{2}\theta }}d\theta$$

and

(6) $$E\left(s\right)=\int_{0}^{\frac{\pi }{2}}\sqrt{1-s^{2}\sin^{2}\theta }d\theta .$$

For every winding of a coil, Equation (2) is calculated and for every combination of two windings, Equation (3)–(6) is calculated. In Figure 4 the cross section of a coil with four windings distributed over two layers is shown. To calculate the self-inductance of this coil for instance, every combination of a set of windings needs to be calculated, as well as the contribution of each individual winding. This can be represented in a matrix like (7) where each $L_{i}$ is obtained using (2) and each $M_{ij}$ is obtained using (3).

(7) $$\left[\begin{array}{cccc} L_{1} & M_{12} & M_{13} & M_{14}\\ M_{21} & L_{2} & M_{23} & M_{24}\\ M_{31} & M_{32} & L_{3} & M_{34}\\ M_{41} & M_{42} & M_{43} & L_{4} \end{array}\right]$$

The mutual inductance is reciprocal; therefore, the contribution of winding *i* on winding *j* is the same as winding *j* on winding *i*. In other terms: $M_{ij}=M_{ji}$. This makes the inductance matrix a symmetrical matrix with the self-inductances on the diagonal. The sum of all elements of the matrix results in the total self-inductance of the coil.

#### 2.1.2. Mutual Inductance Between Two Aligned Coils

**Figure 5:**
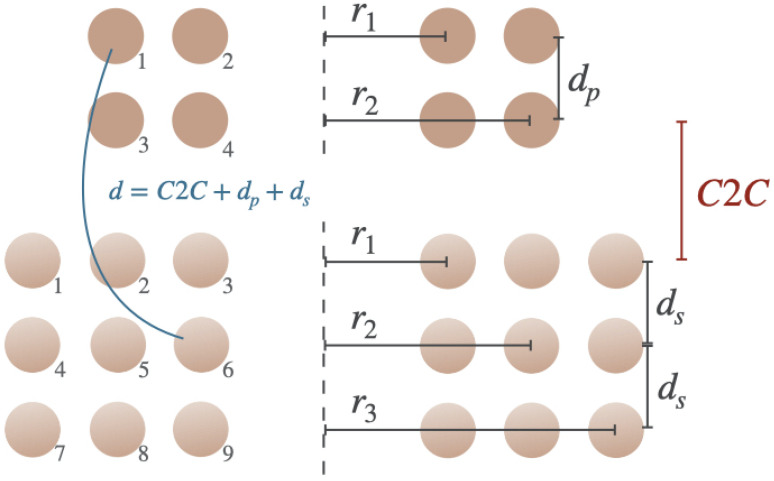
Cross section of two coils coaxially aligned.

To calculate the mutual inductance between two coils that are concentrically aligned like in Figure 5, Equation (3) can again be used. Variables $r_{1}$ and $r_{2}$ are then the different radii of the primary coil $r_{p}$ and the secondary coil $r_{s}$, respectively. The distance *d* is again the distance between two windings but from different coils now. In the case of multiple layers, *d* will take the distance between the layers of the primary coil $d_{p}$, the distance between the layers of the secondary coil $d_{s}$ and the vertical distance between the coils, also called the coil-to-coil distance (C2C), into account as shown in blue in Figure 5. The dimension of the inductance matrix (8) will be *n* by *m*, with *n* as the number of windings of the primary coil and *m* as the number of windings of the secondary coil. The elements in the matrix are the mutual inductances between the primary and secondary coil. $M_{p_{n}s_{m}}$ represents the mutual inductance of winding *m* of the secondary coil on winding *n* of the primary coil. The sum of all elements results again in the total mutual inductance between the coils.

(8) $$\left[\begin{array}{ccccc} M_{p_{1}s_{1}} & M_{p_{1}s_{2}} & \cdots & M_{p_{1}s_{m-1}} & M_{p_{1}s_{m}}\\ \vdots & \; & \ddots & \; & \vdots \\ M_{p_{n}s_{1}} & M_{p_{n}s_{2}} & \cdots & M_{p_{n}s_{m-1}} & M_{p_{n}s_{m}} \end{array}\right]$$

#### 2.1.3. Mutual Inductance Between Two Coils with Lateral and/or Angular Misalignment

**Figure 6:**
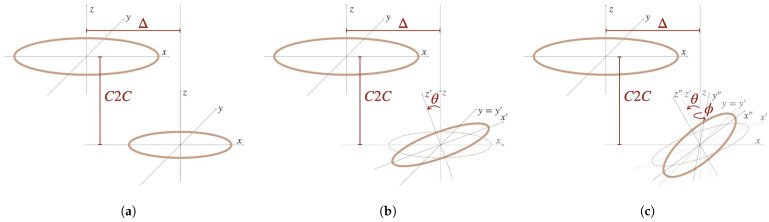
Different misalignments. (**a**) Lateral; (**b**) lateral and lateral angular; (**c**) lateral, lateral angular and longitudinal angular.

Lateral misalignment Δ is the most straightforward to comprehend and to calculate and is shown in Figure 6a. Angular misalignment can be described by three angles, i.e., the Euler angles. The first angle θ represents the latitude angular misalignment. This is the angle in which the secondary coil is rotated around the y-axis, as shown in Figure 6b. The second angle ϕ represents what we call the longitude angular misalignment, defined as the angle in which the secondary coil can be rotated around the z-axis, as shown in Figure 6c. The third angle, the one around the x-axis, is not shown on the figures. As a result of the symmetry of the circles that represents the coils in these figures, this angle is not needed to acquire all possible positions. If the helicity of the spiral coils is taken into account, this angle does need to be known, although its impact on the mutual inductance is rather small.

The challenge is to find a formula like Equation (3) for the mutual inductance that also takes lateral and angular misalignments into account. Different approaches to take these misalignments into account when calculating the mutual inductance between two coils can be found in literature. Two are discussed in the following section.

### 2.2. Literature Review

Two ways of calculating the mutual inductance between two coils with lateral and angular misalignment are presented in this section. One is based on the approach presented by Anele et al. [20] and Babic et al. [21] and the other is on the approach by Liu et al. [22]. At the end of this subsection, one approach will be selected to build a MATLAB implementation.

#### 2.2.1. First Approach (Anele et al. [20] and Babic et al. [21])

The aim of Anele et al. [20] is to re-derive mathematical models to calculate the mutual inductance based on the application of Maxwell’s formula, Neumann’s formula, the Biot–Savart law and magnetic vector potential to more efficient and genaral formulas expressed over elliptic integrals of the first and second kind. Well detailed derivations of Neumann’s formula as presented by Grover [23] result in Equation (9)–(15). The formula calculates the mutual inductance between two circular loops and takes vertical, lateral and angular misalignment into account.

(9) $$M=\frac{2\mu_{0}}{\pi }\sqrt{r_{p}r_{s}}\int_{0}^{\pi }\frac{\left(\cos\theta -\frac{\Delta }{r_{s}}\cos\psi \right)k\left(s\right)}{s\sqrt{V^{3}}}d\psi$$

where

(10) $$k\left(s\right)=\left(1-\frac{s^{2}}{2}\right)K\left(s\right)-E\left(s\right),$$

(11) $$s^{2}=\frac{4\alpha V}{{\left(1+\alpha V\right)}^{2}+\xi^{2}},$$

(12) $$V=\sqrt{1-\cos^{2}\psi \sin^{2}\theta -2\frac{\Delta }{r_{s}}\cos\psi \cos\theta +\frac{\Delta^{2}}{r_{s}^{2}}},$$

(13) ξ=β−αcosψsinθ,

(14) $$\beta =\frac{d}{r_{p}},$$

(15) $$\alpha =\frac{r_{s}}{r_{p}},$$

and K(s) and E(s) the complete elliptic integrals of the first and second kind, respectively, $r_{p}$ is the radius of the primary coil, $r_{s}$ the radius of the secondary coil, θ the lateral angular misalignment, Δ the lateral misalignment, *d* the vertical misalignment and ψ the angle of integration at any point of the secondary coil. Figure 6b is a representation of this case of two coils with vertical, lateral and angular misalignment.

The general formula (9) is split into four cases. All of them are covered, seeing that a fast MATLAB implementation is one of the goals of this research. Using the specific equation for the case in question might result in faster calculation rather than always using the general equation and setting the right parameters to zero.

The first case is one without lateral or angular misalignment. There is only the C2C distance to take into account. In this case V=1 and Equation (9) simplifies to the following formula, Equation (16)–(19):

(16) $$M=\frac{2\mu_{0}}{\pi }\sqrt{r_{p}r_{s}}\int_{0}^{\pi }\frac{\left(1-\frac{s^{2}}{2}\right)K\left(s\right)-E\left(s\right)}{s}d\psi$$

where

(17) $$s^{2}=\frac{4\alpha }{{\left(1+\alpha \right)}^{2}+\xi^{2}},$$

(18) $$\alpha =\frac{r_{s}}{r_{p}},$$

and

(19) $$\xi =\frac{d}{r_{p}}.$$

If α and ξ are filled in and the integral is calculated, Equation (16) results into Equation (3) again.

The second case takes lateral misalignment into account. Equation (9) then results into the following, Equation (20)–(25):

(20) $$M=\frac{2\mu_{0}}{\pi }\sqrt{r_{p}r_{s}}\int_{0}^{\pi }\frac{\left(1-\frac{\Delta }{r_{s}}\cos\psi \right)k\left(s\right)}{s\sqrt{V^{3}}}d\psi$$

where

(21) $$k\left(s\right)=\left(1-\frac{s^{2}}{2}\right)K\left(s\right)-E\left(s\right),$$

(22) $$s^{2}=\frac{4\alpha V}{{\left(1+\alpha V\right)}^{2}+\xi^{2}},$$

(23) $$V=\sqrt{1-2\frac{\Delta }{r_{s}}\cos\psi +\frac{\Delta^{2}}{r_{s}^{2}}},$$

(24) $$\alpha =\frac{r_{s}}{r_{p}},$$

and

(25) $$\xi =\frac{d}{r_{p}}.$$

The third case covers angular misalignment only, resulting in Equation (26)–(31)

(26) $$M=\frac{2\mu_{0}}{\pi }\sqrt{r_{p}r_{s}}\cos\theta \int_{0}^{\pi }\frac{\left(1-\frac{s^{2}}{2}\right)K\left(s\right)-E\left(s\right)}{s\sqrt{V^{3}}}d\psi$$

where

(27) $$s^{2}=\frac{4\alpha V}{{\left(1+\alpha V\right)}^{2}+\xi^{2}},$$

(28) $$V=\sqrt{1-\cos^{2}\psi \sin^{2}\theta },$$

(29) ξ=β−αcosψsinθ,

(30) $$\beta =\frac{d}{r_{p}},$$

and

(31) $$\alpha =\frac{r_{s}}{r_{p}}.$$

When there is no lateral misalignment, there is only one angle θ to take into account, again, because of symmetry. With this angle, all possible positions can be acquired.

The fourth case covers the combination of angular and lateral misalignment and thus describes Equation (9) again. However, what is missing in this formula is the longitude angular misalignment. Equation (9) cannot simply be adapted to incorporate this extra rotation. This case is not extensively reported in the literature.

A study presented by Babic et al. [21], on which [20] is also based, did derive a formula for the mutual inductance between two coils that takes lateral, latitude angular and longitude angular misalignment into account. The authors started from the same mathematical models based on the application of Maxwell’s formula, Neumann’s formula, the Biot–Savart law and magnetic vector potential. The result is shown in Equation (32)–(50).

(32) $$M=\frac{\mu r_{s}}{\pi }\int_{0}^{2\pi }\frac{\left(p_{1}\cos\psi +p_{2}\sin\psi +p_{3}\right)k\left(s\right)}{s\sqrt{V^{3}}}d\psi$$

where

(33) $$k\left(s\right)=\left(1-\frac{s^{2}}{2}\right)K\left(s\right)-E\left(s\right),$$

(34) $$s^{2}=\frac{4V}{A+2V},$$

(35) $$V^{2}=\alpha^{2}\left(\left(1-\frac{b^{2}c^{2}}{l^{2}L^{2}}\right)\cos^{2}\psi +\frac{c^{2}}{l^{2}}\sin^{2}\psi +\frac{abc}{l^{2}L}\sin2\psi \right)+\beta^{2}+\gamma^{2}-2\alpha \frac{\beta ab-\gamma l^{2}}{lL}\cos\psi -\frac{2\alpha \beta c}{l}\sin\psi ,$$

(36) $$A=1+\alpha^{2}+\beta^{2}+\gamma^{2}+\delta^{2}+2\alpha \left(p_{4}\cos\psi +p_{5}\sin\psi \right),$$

(37) $$p_{1}=\frac{\gamma c}{l},$$

(38) $$p_{2}=-\frac{\beta l^{2}+\gamma ab}{lL},$$

(39) $$p_{3}=\frac{\alpha c}{L},$$

(40) $$p_{4}=\frac{\beta ab-\gamma l^{2}+\delta bc}{lL},$$

(41) $$p_{5}=\frac{\beta c-\delta a}{l},$$

(42) $$\alpha =\frac{r_{s}}{r_{p}},$$

(43) $$\beta =\frac{x_{c}}{r_{p}},$$

(44) $$\gamma =\frac{\Delta }{r_{p}},$$

(45) $$\delta =\frac{d}{r_{p}},$$

(46) $$l=\sqrt{a^{2}+c^{2}},$$

(47) $$L=\sqrt{a^{2}+b^{2}+c^{2}},$$

(48) a=sinϕsinθ,

(49) b=−cosϕsinθ

and

(50) c=cosθ.

Anele et al. concludes that the values of the mutual inductance correspond as long as the secondary coil is located inside the boundaries of the primary coil. Once the secondary coil is located outside the primary coil, the values still correspond but become negative and approach zero again for larger values of Δ. The position where the value of the mutual inductance becomes zero is called the transmission efficiency dead-zone (TEDZ). Mohdeb [24] describes the cause of this zone. At a certain misalignment, the flux lines are crossing the coil with the same magnitude but in opposite directions, causing flux cancellation, or the flux lines are not crossing the coil at all because they are parallel with the coil. When the lateral misalignment increases, more flux lines pass through the coil again but in the opposite direction, causing the mutual inductance to become negative. The larger the lateral misalignment, the more the mutual inductance approaches zero again, simply because the secondary coil is too far to still catch the flux lines of the primary coil.

The method of Anele et al. [20] and Babic et al. [21] is applied to coils with only one loop; however, it can be used for coils with multiple loops and layers as well. The vertical distance *d* in Equation (20) then takes the C2C distance into account as well as the distance between the layers of the primary coil $d_{p}$ and the distance between the layers of the secondary coil $d_{s}$. Each of the two coils is approximated by a cluster of concentric circular loops and the mutual inductance is the superposition of the mutual inductance of all these loops. The helicity of the spiral coils is neglected in this way. This assumption is valid as long as the screw pitches, the distance between the concentric turns, are significantly smaller than the radii [22].

#### 2.2.2. Second Approach (Liu et al. [22])

**Figure 7:**
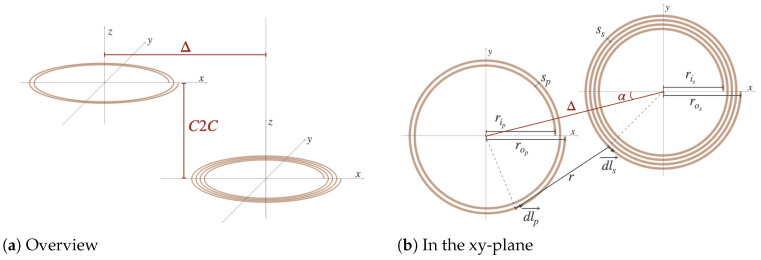
Two Archimedean spiral coils.

Liu et al. [22] do take the helicity of spiral coils into account. They start from Neumann’s formula (52) and the equation of an Archimedean spiral coil (51) in polar coordinates:

(51) $$r=\frac{s}{2\pi }\psi ,\psi_{i}<\psi <\psi_{o}$$

where *r* is the radius, *s* is the screw pitch and ψ the angular coordinate.

(52) $$M=\frac{\mu_{0}}{4\pi }\oint_{C_{1}}\oint_{C_{2}}\frac{\vec{dl_{p}}\vec{dl_{s}}}{r}$$

where $C_{1}$ and $C_{2}$ represent the surface of the coils, $\vec{dl_{p}}$ and $\vec{dl_{s}}$ represent tangential elements at any point on the primary and the secondary coils and *r* is the distance between those two points. In Figure 7a two spiral coils are shown. The lateral misalignment Δ and C2C distance are indicated. In Figure 7b the same coils are shown in the xy-plane in more detail. The primary coil has two turns and the secondary has four. $r_{i}$ is the inner radius, $r_{o}$ is the outer radius, Δ is the distance between the coil centers in the xy-plane (the lateral misalignment) and α is the azimuth angle, i.e., the angle between the x-axis and the straight line connecting the centers. This parameter is introduced to take the helicity and, thus, the starting (and ending point) of the spiral into account.

To calculate $\vec{dl_{p}}$ and $\vec{dl_{s}}$, the coordinates of the points on the spiral need to be derived as Equation (53)–(58):

(53) $$x_{p}=\frac{s_{p}}{2\pi }\psi_{p}\cos\psi_{p}$$

(54) $$y_{p}=\frac{s_{p}}{2\pi }\psi_{p}\sin\psi_{p}$$

(55) $$z_{p}=0$$

(56) $$x_{s}=\frac{s_{s}}{2\pi }\psi_{s}\cos\psi_{s}\cos\theta +\Delta \cos\alpha$$

(57) $$y_{s}=\frac{s_{s}}{2\pi }\psi_{s}\sin\psi_{s}+\Delta \sin\alpha$$

(58) $$z_{s}=C2C+\frac{s_{s}}{2\pi }\psi_{s}\cos\psi_{s}\sin\theta$$

Therefore, the tangential vectors are as Equation (59)–(61):

(59) $$\vec{dl}=\vec{dx}+\vec{dy}+\vec{dz}$$

(60) $$\vec{dl_{p}}=\frac{s_{p}}{2\pi }\left[\left(\cos\psi_{p}-\psi_{p}\sin\psi_{p}\right)\vec{x}+\left(\sin\psi_{p}+\psi_{p}\cos\psi_{p}\right)\vec{y}\right]d\psi_{p}$$

(61) $$\vec{dl_{s}}=\frac{s_{s}}{2\pi }\left[\cos\theta \left(\cos\psi_{s}-\psi_{s}\sin\psi_{s}\right)\vec{x}+\left(\sin\psi_{s}+\psi_{s}\cos\psi_{s}\right)\vec{y}-\sin\theta \left(\cos\psi_{s}-\psi_{s}\sin\psi_{s}\right)\vec{z}\right]d\psi_{s}$$

where ψ goes from $\psi_{i}=r_{i}\frac{2\pi }{s}$ to $\psi_{o}=r_{o}\frac{2\pi }{s}$. The distance between those two vectors is then as follows:

(62) $$\begin{array}{cc} r & =[{\left(\Delta \cos\alpha +a_{2}\psi_{2}\cos\psi_{2}\cos\theta +a_{1}\psi_{1}\cos\psi_{1}\right)}^{2}+{\left(\Delta \sin\alpha +a_{2}\psi_{2}\sin\psi_{2}-a_{1}\psi_{1}\sin\psi_{1}\right)}^{2}\\ & +{\left(C2C-a_{2}\psi_{2}\cos\psi_{2}\sin\theta \right)}^{2}{]}^{\frac{1}{2}}. \end{array}$$

When Equation (60), Equation (61) and Equation (62) are filled in in the Neumann’s Equation (52), this results in Equation (63).

(63) $$M=\frac{\mu }{4\pi }a_{1}a_{2}\int_{\psi_{i2}}^{\psi_{o2}}\int_{\psi_{i1}}^{\psi_{o1}}\frac{\cos\theta \left(\cos\psi_{1}-\psi_{1}\sin\psi_{1}\right)\left(\cos\psi_{2}-\psi_{2}\sin\psi_{2}\right)+\left(\sin\psi_{1}+\psi_{1}\cos\psi_{1}\right)\left(\sin\psi_{2}+\psi_{2}\cos\psi_{2}\right)}{\sqrt{{\left(\Delta \cos\alpha +a_{2}\psi_{2}\cos\psi_{2}\cos\theta +a_{1}\psi_{1}\cos\psi_{1}\right)}^{2}+{\left(\Delta \sin\alpha +a_{2}\psi_{2}\sin\psi_{2}-a_{1}\psi_{1}\sin\psi_{1}\right)}^{2}+{\left(C2C-a_{2}\psi_{2}\cos\psi_{2}\sin\theta \right)}^{2}}}d\psi_{1}d\psi_{2}$$

   With this approach, there are in fact three angles to take into account seeing that the helicity of the spiral coils cancels out the symmetry. However, only θ, as seen in Figure 6b, is covered by the paper. α partially covers the rotation around the z-axis. Partially, this is because it only takes the starting and, thus, the ending point of the coils into account and, as a result, cannot be compared with ϕ in the previous approach. Liu et al. states that only θ has a significant impact on the coupling coefficient.

The conclusion of Liu et al. [22] states that calculating the mutual inductance using (63) results in a smaller error compared to using the traditional method without considering the helicity of the coils. This is especially the case when the screw pitches are larger. Liu et al. also discuss the case without misalignment and only the C2C distance, and the case without the C2C distance and only lateral misalignment.

Seeing that in the application of cochlear implants, small angles of both θ and ϕ are common, and even though they have a small impact on the coupling, it would still be interesting to examine both angles. For this reason, the first approach of Anele et al. [20] is selected for further use.

### 2.3. Methodology

The aim of this research is to build a software tool that quickly calculates the coupling coefficient depending on the parameters entered by the user. COMSOL Multiphysics makes use of finite element analysis. To obtain an acceptable accuracy, many elements are required, which can increase the calculation time immensely. In this research, MATLAB, version R2023a is used; however, any open language such as Python could be used. The different formulas of the first approach Section 2.2.1 discussed in the previous sections are calculated and the coupling coefficient is plotted. The pseudo code of the MATLAB script can be found in Appendix A.

## 3. Results and Validation

To validate the methods used in this paper, the results of the MATLAB scripts are compared with the results of COMSOL simulations. The COMSOL simulations use finite element analysis (FEA) and integrate over the complete spirals.

The coils that were used for validation are inspired by commercially available CI systems. They have an outer radius of 15 mm, a screw pitch of 0.80 mm and a wire radius of 0.2 mm. The primary coil (the transmit coil) has three turns. This results in three concentric circles with radii 15 mm, 14.2 mm and 13.4 mm. The secondary coil (the receive coil) has five turns. Thus, this results in five concentric circles with radii 15 mm, 14.2 mm, 13.4 mm, 12.6 mm and 11.8 mm. These coils are assumed realistic designs for cochlear implants. In Table 1, the calculated self-inductances in MATLAB are shown, as well as the simulated ones in COMSOL and the difference between them. This difference is calculated as a relative error. A relative error is defined as the difference between the approximated result and the exact value, divided by the exact value. The simulated value in COMSOL is assumed to be the exact value. For instance, the relative error of the self-inductance of the primary coil between the results of MATLAB and FEA in COMSOL is calculated in the following way:

(64) $$\delta L_{p}=\frac{MATLAB-FEA}{FEA}*100$$

**Figure 8:**
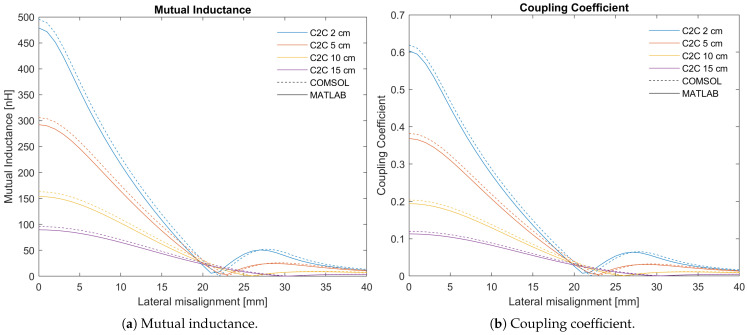
MATLAB calculations vs COMSOL simulations.

**Figure 9:**
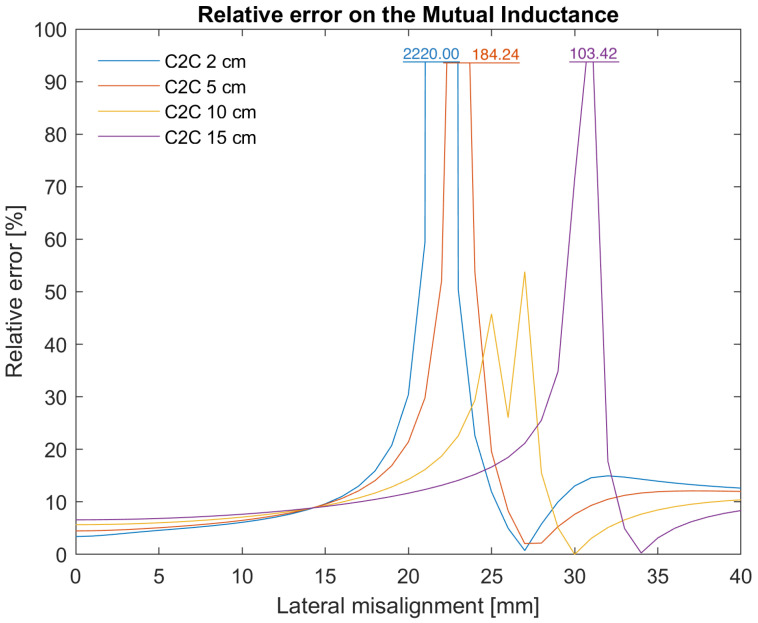
Relative error on the mutual inductance calculated in MATLAB compared to FEA in COMSOL.

The mutual inductance between the two coils for the different C2C distances in function of the lateral misalignment is plotted in Figure 8a. This is just an example to clearly see the difference between MATLAB and COMSOL; there are, of course, two more dimensions of the angular misalignments. The dotted lines are calculated using COMSOL and the solid lines using MATLAB. The mutual inductance is calculated for a lateral misalignment ranging from 0 to 40 mm every 1 mm and a C2C for values of 2 mm, 5 mm, 10 mm and 15 mm. For each data point, the error is calculated using Equation (64). From Figure 8a, it seems that the absolute value of the error is approximately the same, independent of the lateral misalignment or C2C distance. For the relative error, however, this is not the case. The results are plotted in Figure 9. For some C2C distances, the relative errors are significantly exceeding 100%. To make the graph more intuitive, the axis of the relative error is shown until 100%. Exceeding peaks are topped of with an indication of the value this peak reaches.

To be able to put a number on the error of the mutual inductance, the average of the absolute value of all errors is calculated using (65).

(65) $$\delta M=\frac{\sum_{i=0}^{N}|\delta M_{i}|}{N}$$

where $M_{i}$ is the error on the $i^{th}$ data point and *N* is the amount of data points, which are 164 in this case. The average error on the mutual inductance is shown in Table 2, i.e., 27.02% as well as the median, i.e., 8.74%.

The coupling coefficient between the coils for the different C2C distances in the function of the lateral misalignment is plotted in Figure 8b. The graph is similar to the one of the mutual inductance (Figure 8a). This is as expected seeing that the coupling coefficient is the quotient of the mutual inductance and a constant, namely the square root of the product of the self-inductances. There is a small difference between the lines of both plots though, seeing that the self-inductance calculated by MATLAB has a small error compared to the calculations in COMSOL. The error of the coupling coefficient is calculated in the same way as that of the mutual inductance and is shown in Table 2 as well, i.e., 26.56%, together with the median value i.e., 8.10%.

## 4. Discussion

The errors in the self-inductance calculated in MATLAB can be explained by the fact that the spiral coils are approximated by concentric circles, while FEA in COMSOL works with real Archimedean spiral coils. For the mutual inductance, the coils are again approximated by concentric circles while finite element analysis assumes spirals. There is, however, an extra reason for the average error of 27.02%. When looking at Figure 9 there are clearly some outliers. They happen around 20 mm for small C2C distances and move towards 30 mm for larger C2C distances. This point coincides with the moment the mutual inductance approaches zero, the transmission efficiency dead-zone. Calculating the relative error gives a good indication of the scale of the error. However, for very small values going to zero, this is not a good idea as this results in a division by a very small number, causing very large errors. A small error on a large mutual inductance results in a small relative error, while the same small error on a very small mutual inductance results in a very large relative error. This is what happens in the TEDZ when the mutual inductance and the coupling coefficient approach zero. The resolution of the data points also plays a role here. For instance, if the error would only be calculated once every 5 mm lateral misalignment, it could be that the TEDZ is accidentally skipped and as a result the average error would be smaller. It is clear that this is a very critical point and it is useful to know where it is. For error calculations, these outliers increase the average error significantly. Therefore, the median is also calculated and is a better measure of center in this particular case. For the mutual inductance, the median is 8.74%. If the TEDZ would be excluded from the calculation of the average error, a number close to this value would result.

For the coupling coefficient it may be expected that the error is higher than the one of the mutual inductance seeing that *k* is calculated using approximations of the self and mutual inductances, and so the errors propagate through the operations. However, when looking at Table 2 this is not the case. The error becomes smaller, as can be proven with Equation (66)–(68):

(66) $$\delta p=\delta L_{p}+\delta L_{s}$$

(67) $$\delta \sqrt{p}=\frac{1}{2}\delta p$$

(68) $$\delta k=\delta M-\delta \sqrt{p}$$

where δp is the relative error of the product of $L_{p}$ and $L_{s}$.

For the same reason as for the mutual inductance, the median error of the coupling coefficient is also calculated and is again a better measure of the error. The median error has a value of 8.10%.

**Figure 10:**
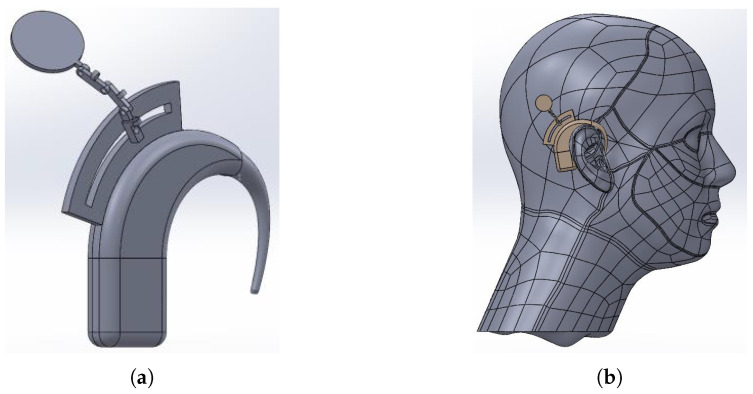
Example of a BTE where the coil stays mechanically in place. (Research in preparation of a master’s thesis). (**a**) The BTE. (**b**) The BTE positioned on the head.

When looking back at the application of a cochlear implant, there are two types of constraints: patient constraints and technology constraints. A first patient constraint is the skin flap thickness (SFT). This forces the C2C distance to be between 1 mm minimally and 12 mm maximally. The lateral misalignment angle is constraint by a second patient constraint, namely the way the coil is implanted and the curvature of the human head. As a result, the angle θ cannot be much larger than 10° or smaller than −10°. A technology constraint is that even though perfect alignment cannot be guaranteed, it is the goal to design a BTE that is restricted in size and therefor restricted in lateral misalignment. Research into different first design options as shown in Figure 10, shows that in worst-case scenarios like heavy exercising, for instance, the lateral misalignment will be 12.9 mm at maximum.

Taking all these constraints into account, the operating region ranges from 1 to 12 mm C2C distance, 0 to 13 mm lateral misalignment and −10° to 10° degrees lateral angular misalignment (the longitude angular misalignment is not constrained). This means that the transmission efficiency dead-zone will lay outside the operating region. So, we can safely say that the average error on the coupling coefficient for the application of a cochlear implant will not be larger than 8.10%. This is confirmed by recalculating the average error for the operating region only, which results in a value of 4.76%.

Apart from the implementation error, it is of course interesting to check if magnet-free implants would in fact be feasible. Looking at the MATLAB generated plots of the operating region, the coupling coefficient is greater than 0.095 for all misalignments. Typically, perfectly aligned conventional cochlear implants with magnets have a coupling coefficient between 0.1 and 0.4. Further research into power and data transfer with a coupling coefficient ranging from 0.095 to 0.1 is thus required. However, this is a very small difference and the coupling coefficient is larger than or equal to 0.1 for the largest part of the operating region. This analysis raises the confidence that magnet-free cochlear implants are indeed feasible.

To conclude, the error on the coupling coefficient that is already rather small, is much less significant compared to the difference in calculation time. The initial goal was to have a way to quickly generate the coupling coefficient. It took 53.7 h to generate all the different misalignments shown in Figure 8a,b using COMSOL on a dedicated server. Using MATLAB on a standard laptop, the same data points were rendered in 23.41 s. When the angular misalignments are taken into account as well, for example, θ and ϕ ranging from −90° to 90° with steps of 15°, it took MATLAB 108 min and 51.77 s. The calculations become a little more complex, seeing that some of these points are physically not possible for the coil dimensions used in this example (e.g., the windings of the transmit and receive coils would intersect). These points should thus be excluded from the calculations. Generating the same data in COMSOL took over 7 days, after which the simulation was prematurely stopped for power saving reasons. It is thus very clear that the MATLAB script can give a quick indication whether there are coil designs that demonstrate potential. If so, further qualitative calculations can still be performed. This results in significant time-saving and it provides an interesting method for initial exploration of broad options and different technologies.

In the future, the second approach discussed in Section 2.2 can be looked into to see if the longitude angular misalignment ϕ can be added and if the error might even be smaller, as long as the calculation time does not increase significantly.

## 5. Conclusions

The aim of this research was to quickly get an indication of the value of the coupling coefficient between two spiral coils depending on the coil-to-coil distance, the lateral misalignment and the latitude and longitude angular misalignment. It was shown that three orders of magnitude improvement in execution time could be achieved for a representative case of four different C2C distances and 41 different lateral misalignments with a median error of 8%. The size of the error is negligible, especially compared to the significant time-saving. With the outcome, the design process of magnet-free inductive links in cochlear implants could speed up significantly. If the MATLAB implementation is used in the operating region of a cochlear implant, i.e., a C2C distance of 1 mm to 12 mm, a lateral misalignment from 0 to 13 mm and a lateral angular misalignment of maximum 10°, the error is further reduced to below 5% due to the absence of the transmission efficiency dead-zones in this region. Furthermore, this TEDZ can be accurately located, allowing to make quick feasibility estimations of different coil setups or applications.

In the future, other approaches to implement a MATLAB script can be looked into to possibly obtain an even smaller error and an even faster script.

## Figures and Tables

**Table 1:** MATLAB results compared to FEA in COMSOL for the self-inductance.

	MATLAB [nH]	FEA [nH]	$\delta_{M\text{-}\mathrm{FEA}}$ [%]
$L_{p}\left(TX\right)$	537.88	540.13	0.42
$L_{s}\left(RX\right)$	1169.55	1185.90	1.38

**Table 2:** MATLAB results compared to FEA in COMSOL for the mutual inductance and the coupling coefficient.

	MATLAB [nH]	FEA [nH]	Average $\delta_{M-\mathrm{FEA}}$ [%]	Median $\delta_{M-\mathrm{FEA}}$ [%]
*M*	Figure 8a	Figure 8a	27.02	8.74
*k*	Figure 8b	Figure 8b	26.56	8.10

## Data Availability

The original contributions presented in this study are included in the article. Further inquiries can be directed to the corresponding author.

## Abbreviations

    The following abbreviations are used in this manuscript:

BTE	Behind-the-Ear
CI	Cochlear Implant
C2C	Coil-to-Coil
FEA	Finite Element Analysis
MRI	Magnetic Resonance Imaging
SFT	Skin Flap Thickness
TEDZ	Transmission Efficiency Dead-Zone

## Appendix A

**Algorithm A1:** Self-inductance of a coil: self_inductance
**Input**: - Array of radii *r* - Wire radius *a* - Number of layers *l* - Distance between the layers *d* **Output**: - Self-inductance *I* **Initialisation**: Set *N* = size of *r* Set turnsPerLayer = $\frac{N}{l}$ Preallocate inductanceMatrix, matrix *N*x*N* Calculate self-inductance: $I=\sum_{i=0,j=0}^{N-1,N-1}inductanceMatrix$
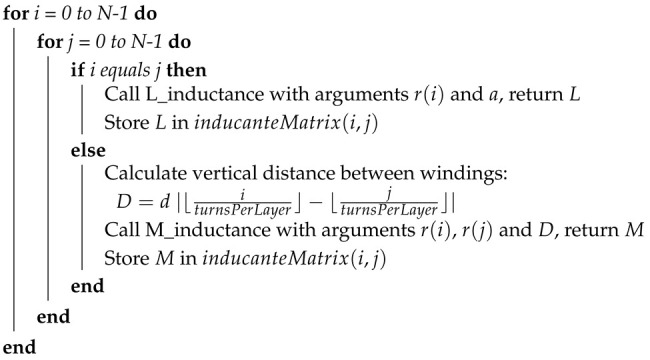

**Algorithm A2:** Inductance for one circular winding: L_inductance
**Input**: - Radius *r* - Wire radius *a* **Output**: - Inductance *L* **Initialisation**: Set $\mu =4\pi 10^{-7}$ Calculate inductance: $L=\mu \;r\;\log\left(\frac{8\;r}{a}-1.75\right)$

**Algorithm A3:** Mutual inductance between two windings: M_inductance
**Input**: - Radius of winding 1 $r_{1}$ - Radius of winding 2 $r_{2}$ - Distance between the windings *d* **Output**: - Mutual inductance *M* **Initialisation**: Set $\mu =4\pi 10^{-7}$ Set $s=\sqrt{\frac{4r_{1}r_{2}}{{\left(r_{1}+r_{2}\right)}^{2}+d^{2}}}$ Call ellipke with arguments $s^{2}$ and $10^{-7}$, return *K* and *E* Calculate mutual inductance: $M=\mu \;\sqrt{r_{1}r_{2}}\left[\left(\frac{2}{s}-s\right)K-\frac{2}{s}E\right]$

**Algorithm A4:** Mutual inductance between two coils: mutual_inductance
**Input**: - Array of radii of the primary coil $r_{p}$ - Number of layers of the primary coil $l_{p}$ - Distance between the windings of the pimary coil $d_{p}$ - Array of radii of the secondary coil $r_{s}$ - Number of layers of the secondary coil $l_{s}$ - Distance between the windings of the secondary coil $d_{s}$ - C2C distance between the two coils *c* **Output**: - Mutual inductance between the two coils MI **Initialisation**: Set *N* = size of $r_{p}$ Set $turnsPerLayer_{p}$ to $\frac{N}{l_{p}}$ Set *M* = size of $r_{s}$ Set $turnsPerLayer_{s}$ to $\frac{M}{l_{s}}$ Preallocate inductanceMatrix, matrix *N*x*M* Calculate mutual inductance: $MI=\sum_{i=0,j=0}^{N-1,M-1}inductanceMatrix$
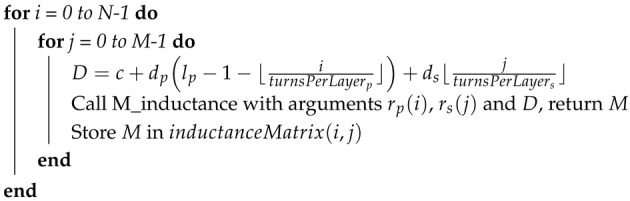

**Algorithm A5:** Mutual inductance between two coils with lateral misalignment: mutual_inductance_Δ
**Input**: - Array of radii of the primary coil $r_{p}$ - Number of layers of the primary coil $l_{p}$ - Distance between the windings of the primary coil $d_{p}$ - Array of radii of the secondary coil $r_{s}$ - Number of layers of the secondary coil $l_{s}$ - Distance between the windings of the secondary coil $d_{s}$ - C2C distance between the two coils *c* - Lateral misalignment between the coils Δ **Output**: - Mutual inductance between the two coils MI **Initialisation**: Set $\mu_{0}=4\pi 10^{-7}$ Set *N* = size of $r_{p}$ Set $turnsPerLayer_{p}$ to $\frac{N}{l_{p}}$ Set *M* = size of $r_{s}$ Set $turnsPerLayer_{s}$ to $\frac{M}{l_{s}}$ Preallocate inductanceMatrix, matrix *N*x*M* Calculate mutual inductance: $MI=\sum_{i=0,j=0}^{N-1,M-1}inductanceMatrix$
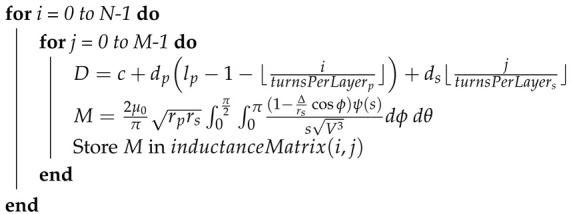

**Algorithm A6:** Coupling coefficient between two coils with lateral misalignment
**Input**: - Array of radii of the primary coil $r_{p}$ - Wire radius of the primary coil $a_{p}$ - Number of layers of the primary coil $l_{p}$ - Distance between the windings of the primary coil $d_{p}$ - Array of radii of the secondary coil $r_{s}$ - Wire radius of the secondary coil $a_{s}$ - Number of layers of the secondary coil $l_{s}$ - Distance between the windings of the secondary coil $d_{s}$ - Array of C2C distances between the two coils *c* - Array of lateral misalignments between the coils Δ **Output**: - Matrix *K* of coupling coefficient for a range of C2C and Δ between the two coils **Initialisation**: Set size_c = size of *c* Set size_Δ = size of Δ Call L_inductance with arguments $r_{p}$, $a_{p}$, $l_{p}$ and $d_{p}$, return $L_{p}$ Call L_inductance with arguments $r_{s}$, $a_{s}$, $l_{s}$ and $d_{s}$, return $L_{s}$ Preallocate K, matrix size_c x size_Δ
